# Next College Session

**Published:** 1854-11

**Authors:** 


### THE NEXT COLLEGE SESSION.

This will commence on the first of November next, and close on the first of March ensuing. This will prove a slight increase in the length of the session. We are of the opinion that the sessions in all our schools should be extended and this appears to be a prevailing sentiment at this time. There are very many reasons for the adoption of such a course which we will not now discuss.

From present indications the class of next winter will outnumber even that of the last, as we are every day in receipt of notices of intention. Those intending to be present will do well to give timely notice (if they have not already done it,) to the Dean to that effect, for many good and substantial reasons.

We have assurances that the supply of books will be equal to the wants of the class, there being now two large book stores at this time in the city, where they can be obtained at as reasonable prices as any other city of the west. The accommodations for board will be ample and comfortable.

Those desiring certificates of Scholarship will call upon the District and County Judges and members of the Senate and Legislature, who are now or will be supplied with them.

This is a State institution and the present arrangement is for the especial benefit of the young men who desire to complete their medical education. Every intelligent citizen with whom we have conversed, have spoken in the warmest terms of commendation of the arrangement, and do not hesitate to say that it will redound to the best interests of the profession and of community. Every liberal and just man has unequivocally declared it to be an element of scientific advancement such as to constitute an object of just pride by every good citizen, and should be cherished and sustained, as it makes such liberal provision for the medical education of our young men at home at a trifling expense to them. With such men, above narrow and selfish views, the great increase in the numbers of the class of last winter was a source of sincere gratulation and awakened in their minds the liveliest hopes and the earnest wishes for the future well-being of an institution promising so much for the public good. We have numerous letters in our possession from gentlemen, in and out of the profession, encouraging us to continued and persevering effort, and assuring us of their warmest sympathy and promises of their efficient aid in our efforts to promote the prosperity and well-being of the school. To all such, we tender our heart-warm gratitude and will be remembered by us in future time. After all this it would hardly be supposed there would be one found so lost to a sense of the public good who would use his influence to persuade young men not to avail themselves of the benefits so liberally held out to them, and yet it is even so, as the history of last winter will abundantly testify. It however availed nought, as all such unhallowed efforts do ultimately fail. This will suggest a wise caution to others not to be beguiled to their own injury merely to gratify an evil propense and vindictive spleen.

All operations performed before the class will be gratuitous, and we take this occasion to give an assurance that this will be adhered to and if heretofore at any time a different course was pursued, it was done without the *knowledge or consent* of the Faculty as such. During last winter no such charge was make in any case.

